# PREVALENCE AND DETERMINANTS OF EATING DISORDER RISK AMONG TUNISIAN UNIVERSITY STUDENTS

**DOI:** 10.1192/j.eurpsy.2023.1794

**Published:** 2023-07-19

**Authors:** M. Turki, F. Jemil, H. E. Mhiri, A. Samet, F. Bennaceur, N. Halouani, S. Ellouze, J. Aloulou

**Affiliations:** 1 Psychiatry “B” department, Hedi Chaker university hospital, Sfax, Tunisia

## Abstract

**Introduction:**

The transition to college life can be a stressful period for young adults, and coping strategies can involve changes in eating behaviors.

**Objectives:**

The aim of this study was to assess the prevalence and correlates of eating disorder risk among Tunisian university students.

**Methods:**

We conducted a cross-sectional descriptive and analytical study among 144 university students in Tunisia. Data were collected using an online questionnaire spread throughout social media (Facebook), using the Google Forms® platform.

Attitudes, feelings and behaviors related to eating were measured using “Eating Attitudes Test” (*EAT-26*) in order to assess the eating disorder risk.

**Results:**

The mean age of our population was 23.38±3.27 years. More females (73.6%) than males (26.4%) participated in the study. Among them, 10.4% were followed for chronic somatic disease while 11.1% suffered from mental illness. Tobacco, alcohol and cannabis use was noted respectively in 12.5%, 3.5% and 3.5% of cases.

The mean score EAT-26 was 20.45. According to this scale, 32,6% of participants were at high risk of eating disorders.

EAT-26 scores were higher in females (21.23) than males (16.95%), without a significant relationship.

Users of psychoactive substances were more likely to present higher EAT-26 scores (p=0.012), especially the use of alcohol (p=0.005) and weed (p=0.024).

EAT-26 scores were significantly higher among students with a prior history of depression.

**Conclusions:**

Our study highlighted a high prevalence of eating disorder risk in university students. Implementation of public health policies are required, and psychological intervention and health awareness programs would effectively prevent the eating disorder risk.

**Disclosure of Interest:**

None Declared

